# Mutant p53 as a Regulator and Target of Autophagy

**DOI:** 10.3389/fonc.2020.607149

**Published:** 2021-02-03

**Authors:** Yong Shi, Erik Norberg, Helin Vakifahmetoglu-Norberg

**Affiliations:** Department of Physiology and Pharmacology, Karolinska Institutet, Stockholm, Sweden

**Keywords:** autophagy, chaperone-mediated autophagy, cancer, mutant p53, *TP53*

## Abstract

One of the most notoriously altered genes in human cancer is the tumor-suppressor *TP53*, which is mutated with high frequency in more cancers than any other tumor suppressor gene. Beyond the loss of wild-type p53 functions, mutations in the *TP53* gene often lead to the expression of full-length proteins with new malignant properties. Among the defined oncogenic functions of mutant p53 is its effect on cell metabolism and autophagy. Due to the importance of autophagy as a stress adaptive response, it is frequently dysfunctional in human cancers. However, the role of p53 is enigmatic in autophagy regulation. While the complex action of the wild-type p53 on autophagy has extensively been described in literature, in this review, we focus on the conceivable role of distinct mutant p53 proteins in regulating different autophagic pathways and further discuss the available evidence suggesting a possible autophagy stimulatory role of mutant p53. Moreover, we describe the involvement of different autophagic pathways in targeting and degrading mutant p53 proteins, exploring the potential strategies of targeting mutant p53 in cancer by autophagy.

## Introduction

Today, the tumor suppressor protein, p53, is not only known for its *bona fide* function as a transcription factor that controls a network of responsive genes during various cellular stress to ensure genomic stability and fidelity, but also for its key regulatory function in major signaling and metabolic adaptation, beyond preventing tumorigenesis (1–3). Correspondingly, *TP53*, is one of the most notoriously altered genes and tumor-associated p53 mutations are found with high frequency in more human cancers than any other tumor suppressor gene (4, 5). While mutations are found all over the *TP53* gene (5), most common alterations rise from single-base protein-altering substitutions in the coding region with heavy mutational pressure of particular nucleotides (6, 7). These commonly occurring missense mutations cluster in the DNA-binding domain with often diminished ability to bind specific DNA recognition sequences (2). Consequently, the primary outcome of *TP53* mutations is loss of the wild-type ability to transactivate canonical p53 target genes, which provides a fundamental advantage for cancer development. A good example of this is the mutant p53^R248^, which amplifies the pro-survival effects of wild-type protein by maintaining the expression of *CDKN1A* gene, resulting in an ability to survive glutamine and serine starvation, while this mutant no longer is able to induce cell death or senescence (8). Furthermore, unlike mutations in other tumor suppressors, the vast majority of *TP53* missense mutations result in expression of stable, full-length mutant variants where cancer cells acquire selective advantages by retaining these form of the protein (9, 10). Beyond exerting dominant repression over the wild-type counterpart due to loss of heterozygosity, some mutants might exert new malignant abilities distinct from those simply caused by the loss of the wild-type function (11, 12). Such phenotypes, described as mutant p53 gain of oncogenic function(s) (GOFs) (9, 13), include increased cell proliferation, migration and invasion as well as anti-apoptotic functions, which actively contribute to various stages of tumor progression (9, 14–17). This has led to the assumption that, during development of certain tumor types, mutations leading to the expression of missense proteins appears prone to be selected for over the null mutations. In support of this, patients carrying tumors with mutant p53 proteins display higher oncogenic potential, poor prognosis, poor response to chemotherapy and accelerated tumor recurrence compared to patients with p53 null tumors (13, 14). The enhanced oncogenic GOF potential of p53 mutants, beyond the loss of p53 function, is best exemplified by studies using mice with point mutations (p53R270H/- and p53R172H/) as models for the human Li-Fraumeni syndrome, which is an autosomal dominant inherited cancer susceptibility disorder resulting from germline mutations in the *TP53* gene (14, 18–21). These studies have demonstrated that knock-in mice of mutants corresponding to human R175H and R273H develop distinct tumor spectra with high frequency of metastasis, contrary to that observed in mice with p53 deletion, signifying the gain of function of the mutant p53 proteins.

Since the discovery of oncogenic feature of mutant p53 proteins, there has been a steady increase in the number of described diverse GOFs in many cancer types. This has led to reported phenotypic characteristics, culminating in several mechanisms suggested as basis for the gained mutant specific activities (4, 22, 23). A well-recognized mechanism of gained mutant p53 function is its interaction with other transcription factors (4, 24), causing profound alterations in the cancer cell transcriptome and the resulting proteome. However, there is no consensus on the molecular definition of most aspects of mutant p53 GOF(s) and their consequential effects. Distinct mutation type-dependent oncogenic activities still remain to be defined. In addition, recent methodological advances, such as integrated ‘-omics’ and p53 saturation mutagenesis screens, present new insights into the clinical outcomes in patients with myeloid malignancies, where no evidence of GOF for *TP53* missense mutation could be found. Instead clonal selection, driven by the loss of canonical p53 function or the dominant-negative effect that reduced the tumor suppressor activity of wild-type p53, was suggested as the most prominent factor in the selective advantage associated with p53 mutations (25, 26). These observations argue for a fitness advantage in certain human tumors harboring missense mutations rather than the acquirement of additional functions, suggesting that GOF may be context and tumor type dependent.

It is further important to consider that although few missense substitutions (Arg175, Gly245, Arg248, Arg273, and Arg282) account for about 30% of all *TP53* mutations, there are more than 1,500 types of p53 mutations reported in various cancer types (http://p53.iarc.fr/), and different mutant variants are frequently detected in different human cancers (6). Yet, not all mutant proteins accumulate at high levels in tumor cells, although such stabilization seems key for mutant p53 proteins to orchestrate its oncogenic behavior (2, 27). Further, growing evidence from *in vitro* studies as well as animal models signifies that the oncogenic activities of mutant p53 variants are heterogeneous and can vary with the tissue type and the genetic background of the cells (28, 29). This predicts that tissue-selective mutational activity would manifest as tissue-selective enrichment of select *TP53* mutations. In fact, 25 different *TP53* mutation were found to be overrepresented in specific tumor types (26). Accordingly, it has become evident that not all p53 mutants are equal or behave alike and the prognostic impact of *TP53* mutations are diverse (30, 31). Therefore, generalizations about mutant p53 may not be relevant. Instead, the discrepancy of the type of mutant is important, not only as a conceptual distinction for their unique oncogenic abilities, but also for the clinical implications including diagnosis, surveillance and therapy.

For the purpose of this review, we will focus on describing and discussing the considerable distinct effects of mutant p53 proteins may exert on autophagy, although other mutant p53 activities may affect different aspects of tumor biology. Autophagy is a fundamental catabolic process by which eukaryotic cells digest macromolecules and damaged organelles in the lysosomes. A well-defined positive regulatory role of wild-type p53 on autophagy with resulting counteracting autophagy inhibitory effect caused by *TP53* mutations, is widely recognized and beyond the scope of this review. Instead, the central focus will be on what we currently know about the conceivable roles of distinct mutant p53 proteins in regulating different autophagic pathways. Further, given that alterations in autophagy activity might vary in different types and stages of tumors, we will elaborate on the emerging rationale that the functional effects of distinct mutant p53 proteins on autophagy may also differ. Given that autophagy is tightly connected to dynamic changes in metabolism, we discuss the concept that in certain conditions cancer cells with mutant p53 may favor instead of counteract autophagy. Furthermore, it is well known today that autophagic pathways are reported to mediate the stability of mutant p53 proteins. In the following parts, we will describe the involvement of different autophagic pathways in controlling the cellular level and degradation of mutant p53 proteins as well as the potential therapeutic strategies for targeting mutant p53 in cancer by various autophagic pathways.

## Autophagy-Lysosomal Pathways

Autophagy is a highly conserved homeostatic recycling process, where it functions to mediate the degradation of cellular macromolecules, damaged organelles or internalized pathogens in the lysosomes (32, 33). Under normal physiological conditions, autophagy is maintained at basal level, however, by responding to perturbations in the extracellular environment, e.g. when encountering nutrient deficiency, cells tune the autophagic flux to meet intracellular metabolic demands (34). Thus, beyond the fundamental significance for cellular quality control purposes and the maintenance of cellular and organismal homeostasis, activation of autophagy provides cells with cytoprotective and metabolic adaptations under stress (33–37). Its timely regulation is, therefore, finely controlled by numerous proteins. Dysregulation of autophagy with subsequent altered protein degradation and cellular metabolism, has severe consequences related to several pathophysiological conditions, such as cancer, infection, autoimmunity, inflammatory diseases, neurodegeneration and aging (38).

Multiple routes of degradation through autophagy coexist in mammalian cells that differ in the delivery mechanisms and target specificity, but converge on the same degradation site - the lysosomes (39). Beyond macroautophagy (MA), usually referred to as autophagy, which is the most extensively studied and well characterized type (39), micro- (MI) and chaperone-mediated autophagy (CMA) pathways, are key components of the cellular machinery that play important roles for lysosome-mediated protein degradation (40–42). MA is a multistep process with a nonselective seizing of cytosolic cargo or in a selective fashion that vary in target specificity and induction conditions. It involves the sequential formation of a double-membrane structure, the phagophore that ultimately fuses with lysosomes to degrade sequestered cargos *via* the activity of hydrolases in autolysosomes (43, 44). While Autophagy-related (Atg) proteins act on the *de novo* synthesis and accompanying elongation and closure of the autophagosomes that engulf the cytosolic cargo during MA (45, 46), MI involves the direct uptake of cargo material by the lysosomal or vacuolar membrane and is suggested to occur by either lysosomal protrusion, invagination or with endosomal invagination (41, 47). CMA, on the other hand, applies to select proteins with a pentapeptide motif related to KFERQ that is recognized by the heat shock cognate 71 kDa protein (Hsc70 (also known as HSPA8)) and co-chaperones (48). This interaction forms a chaperone complex that enables the translocation of the cargo protein into the lysosomal lumen *via* binding the lysosomal receptor, lysosome-associated membrane protein 2A (LAMP-2A) (49).

Regardless of the delivery system, the cargo of the autophagic pathways are digested by the lysosomal hydrolases and engendered building blocks are shuttled back to be reused for biosynthesis of macromolecules (50). In this way, autophagy acts as an important internal source of cellular energy through self-degradation process. Hence, the engagement of autophagic pathways confer stress resistance and sustain cell survival that benefit tumor cell growth, especially in nutrient scarce or hypoxic conditions (51). Furthermore, MA may play a critical role in tumor microenvironment and has been proposed to promote tumor dormancy (52), where cancer cells remain in a quiescent state with the potential to relapse. Consequently, autophagy is exploited by cancer cells and malignant tissues often exhibit altered MA activity (53–55), displaying autophagy addiction to sustain stress resistance. Therefore, inhibition of autophagic flux after induction of pro-survival autophagy has been suggested as a strategy to sensitize multiple human cancer types to chemotherapy. However, the role of MA in carcinogenesis is context dependent with reports indicating both pro-tumorigenic and tumor-suppressive roles (56). As a tumor-suppressing mechanism in early-stage carcinogenesis, autophagy dampens inflammation and promotes genomic stability (57). The direct evidence comes from studies using mouse models with genetic knockout of canonical autophagy-related genes, including *ATG5*, *ATG7*, and *BECN1* where impaired autophagy accelerates tumorigenesis in animals (58). However, once a tumor has been established, the nature of autophagy switches and many aggressive tumors acquire reliance on autophagy for growth and survival (51, 59). Thus, in spite of the dual role of autophagy in cancer development and progression differs depending on the genetic context, type of cancer and tumor stage, it is well established today that autophagy is frequently altered in human cancers, with its activation regarded as one of the characteristic key features that contributing to malignant development. In fact, the limited penetrance of mutations in most autophagy genes across human tumors indicates that many human cancer types preserve autophagy function (60), where several well-established oncoproteins and tumor-suppressors whose depletion or mutation promote tumor formation have emerged as eminent regulators of autophagy. In addition, accumulating evidence now also supports a regulatory role for selective autophagy, including mitophagy and non-MA pathways, in human cancer (61, 62). Although CMA was initially suggested to display pro-tumorigenic functions (63), anti-tumor role for CMA is also proposed under physiological conditions in non-transformed cells (61, 64). Further, subsequent studies have demonstrated that CMA plays a more complex and context-dependent role, where cancer cells from different tissues and tumor stages may display varying CMA activity (62). Moreover, growing number of studies provide new insight as to how increased CMA activity can be beneficial for promoting the degradation of proteins displaying dominant oncogenic pro-survival activities in cancer cells (37, 65–68). However, few studies are conducted to assess the therapeutic impact of CMA activation in cancer, thus CMA-based treatment options in humans remain speculative. This is mainly due to the lack of potent chemical modulators of this process and limitation in functional CMA analysis, which mainly rely on expression levels of the known CMA component, LAMP-2A. Accordingly, while defining the major cancer-related pathways, beyond oncogenic signaling that affect autophagy and control tumorigenesis is important, the regulation and roles of selective and non-MA autophagy, such as CMA and MI, in cancer still needs further investigation, thus the subject of this review will be mainly focused on mutant p53 and MA.

## Wild-type p53 – Dual Role in Macroautophagy Regulation

In the past decades, the mechanisms governing regulation of autophagy has been intensively investigated and the impact of p53, mainly on MA, is well described by several groups with detailed mechanisms uncovered. Collectively, the action of wild-type p53 as a pro-autophagic factor in human cancer cells is reflected by its transcriptional activity on a wide range of downstream target genes with autophagy regulatory effects that diverge on cellular functions, including; a) stimulating the nutrient energy sensor AMPK (AMP-activated protein kinase) (AMPK β1/β2 subunits, Sestrin1/2) (69, 70), b) inhibiting the signaling of mTOR (mechanistic target of rapamycin) (*TSC2*, *IGF-BP3*, *REDD1*) (71, 72), c) suppression of PI3K (phosphatidylinositol-3-kinase) activity (*PTEN*), d) promoting the expression of the MA core machinery (*ULK1*, *ULK2*, *ATG7*) (73, 74), e) transactivating *DRAM1* (damage-regulated autophagy modulator 1) and splice variants that effects several stages of autophagy (75), f) upregulation of *HIF-1* (hypoxia inducible factor 1), g) inducing regulators (Isg20L1 and HSF1 (heat shock transcription factor 1) that in turn transactivates autophagy related genes (*ATG7*) (76), h) induction of *TGM2* (transglutaminase 2) which promotes autophagic flux by enhancing autophagic protein degradation and autolysosome clearance (77), i) interfering with the inhibitory interactions between Beclin-1 and Bcl-2 family proteins (incl. Bcl2/Bcl-XL, Bad, Bax, BNip, Mcl-1, Puma) by their direct transcriptional up or down regulation or through DAPK1 (Death-Associated Protein Kinase 1) activation, alternatively by DAPK1 mediated MAP1B interaction (78), and direct physically interacting with Bcl-XL and the p53-regulated human tumor suppressor protein p14ARF [detailed reviewed in (79, 80)]. These pro-autophagic functions of wild-type p53 are most likely credited to its tumor suppressor role under conditions of hypoxia, starvation or DNA damage, by which induction of MA assists to cope with different kind of cellular stress to prevent cell damage and maintain cellular integrity. This is further in line with the involvement of wild type p53 in several signaling pathways that promote autophagy, including MAPK (mitogen-activated protein kinase) family proteins, such as ERK (extracellular signal-related kinase) and JNK (c-Jun N-terminal kinase).

However, beyond these established pro-autophagic functions, wild-type p53 can also counteract autophagy. This inhibitory role is often attributed to the cytosolic pool of p53 under normal growth conditions, connected to G0/G1 phases of the cell cycle, and shown to be mediated through both transcription-dependent, but mainly -independent manner, involving; a) inhibition of the AMP-dependent kinase and thereby activating mTOR (81), b) induction of TIGAR (TP53-induced glycolysis and apoptosis regulator) regulating glycolysis and cellular ROS levels (82), c) transcriptional regulation of micro RNAs (miRs) (*miR-34a* and *miRs-34a/34c-5p* that targets *ATG9A* and *ATG4B*, respectively) (83, 84), d) interaction with Beclin-1 that subsequently facilitates its ubiquitination and proteasome-mediated degradation (85, 86), and e) direct molecular association with RB1CC1/FIP200, a mammalian protein homologous to Atg17 (87), and f) by reducing the accumulation of double stranded RNA and activation of PKR (protein kinase RNA-activated) (88).

Conclusively, these observations have led to the current notion that the action of wild-type p53 on MA is complex, highly context dependent, dictated by the cellular microenvironment and stress condition, along with the cell cycle progression and subcellular distribution of p53 that exert dual roles in autophagy regulation. In support of this, cumulative evidence shows that nuclear wild-type p53 can promote mitophagy by transactivation of *PRKN* (Parkin), a key effector of this selective autophagy, involved in degradation of impaired mitochondria (89). Cytosolic p53, on the other hand, inhibits mitophagy *via* direct binding to Parkin, preventing its translocation to the damaged mitochondria that cannot be removed by mitophagy (89). Accordingly, these findings are not only confirmative of the counteractive roles of p53 in autophagy regulation, but also indicative of the involvement of p53 in other autophagy pathways, beyond MA. However, despite the studies exemplified above, there are still important pending questions about the detailed molecular mechanisms that govern the role of p53 in MA. Further, the potential contribution, regulatory role, and the physiological importance of p53 in other selective macroautophagy, microautophagy or CMA is yet to be explored.

## Mutant p53 as a Regulator of Autophagy

### The Effect of p53 Mutant Proteins on Autophagy

Given that impairment of the wild-type function with predominant pro-autophagic role is provoked by *TP53* mutations, it is expected that mutant proteins can reshape the wild-type-mediated outcomes on autophagy. Accordingly, the current accepted view is that mutant p53 displays a suppressive role in autophagy. This was initially illustrated by the assessment of the effect of ectopically overexpressing 22 different p53 mutant variants on the autophagy in p53 null colon cancer cells (90). Reintroduction of some p53 mutants, including p53^A161T, S227R, E258K, R273H/L,R273L^, but not the p53^P151H,R282W^, exhibited high correlation with efficient suppressive capacity on basal MA. However, the expression of other mutants, including p53^P98S,K120D,V143A,R175C,R175D,R175H,R175P, R181H,L194F,S227K,G245C,R248L,R248W, R249S,R280K^, displayed no or less suppressive effects, or in some cases even enhanced MA. This led to the awareness that certain p53 mutants may exert negative effects on autophagy. A shared feature of these mutants, including p53^A161T, S227R, E258K, R273H/L,R273L^, was shown to be their cytoplasmic localization, most likely with a loss-of-function to promote transactivation-dependent stimulation of autophagy (90). In support of this, it was later shown that the p53^R175H^ or p53^R273H^ mutants indeed suppress the formation of autophagic vesicles and their fusion with lysosomes through the transcriptional repression of key downstream p53 responsive autophagy related genes, as *BECN1*, *DRAM1*, *ATG12*, as well as *TSC2*, *SESN1/2* and *P-AMPK*, resulting in the autophagy blockage (91, 92). Correspondingly, the knockdown of these mutants in cancer cells cause augmented autophagy by affecting signaling at various phases of the autophagic process with a concomitant stimulation of mTOR signaling. However, it should be noted that both p53 deletion and missense mutations can substantially affect the mTOR signaling, where an elevated association of Rheb with lysosomal membranes promote active mTORC1 complexes (92).

The autophagy inhibitory role of mutant p53 proteins was further ascribed to transcriptional-independent actions. Some p53 mutants, as p53^R175H,L194F, R273H^, were unable to form complexes with endogenous Bcl-2 or Bcl-XL, unlike the wild-type. This loss-of wild type function abolishes the capacity to interact, thus cancer cells bearing mutant p53 sustain the inhibitory interactions between Beclin-1 and Bcl-2 family proteins (93). Further, through mTOR stimulation, the aforementioned mutants also convey negative effects on Beclin-1 expression and phosphorylation, thus suppress the functionality of Beclin-1 in autophagy. Likewise, less directly through mTOR stimulation, the p53^G199V^ mutant was demonstrated to gain regulatory function on STAT3 phosphorylation (94), with subsequent transcriptional activation of HIF-1 suggested to contribute to autophagy inhibition. In fact, several multiple mechanisms by which mutant p53 can stimulate HIF-1 have been identified. These includes increased cellular reactive oxygen species (ROS), resulting from less efficient oxidative phosphorylation, or by interference with the binding of HIF-1α to the ubiquitin-protein ligase Mdm2 in hypoxic conditions. However, the functional role of HIF-1 and hypoxia-related genes in autophagy regulation awaits further investigation.

Moreover, by engaging in protein-protein interactions with other transcription factors as a GOF, some cancer-associated p53 mutants were shown with capability of blocking autophagy indirectly by activating several growth factor receptors, such as TGFBR, EGFR, IGFR (95), contributing to sustained active PI3K/Akt/mTOR signaling that subsequently repress autophagy. In breast cancer cells a direct correlation between mutant p53^R273H^ and Akt phosphorylation was demonstrated. Akt, in turn, propagates the effect on its direct downstream target mTOR. Taken together, regulation of the mTOR activity by either constitutive blockage of AMPK signaling or through alternative routes, appears to represent a crucial signaling that occur in cancer cells bearing mutant p53 (91, 92). Thus, regardless of the transcriptional dysregulation or GOF mediated protein-protein interaction, an important implication of these findings is that the autophagy suppressive role of mutant p53 seems mainly to merge on the canonical AMPK-mTOR signaling.

### The Impact of p53 Mutants on Autophagy Through Metabolic Changes

A defining hallmark of cancer is uncontrolled cell proliferation, which is initiated once cells have accumulated adaptations in pathways that control metabolism and proliferation (96, 97). Metabolism provides the energetic and biosynthetic demands of rapid proliferation. Beyond a high glycolytic activity, the most common metabolic alteration in malignancies, rapidly proliferating cancer cells further display a sustained mitochondrial oxidative phosphorylation, as the tricarboxylic acid (TCA) cycle intermediates are important precursors for the synthesis of amino acids, lipids and nucleotides (96–99).

**Figure 1:**
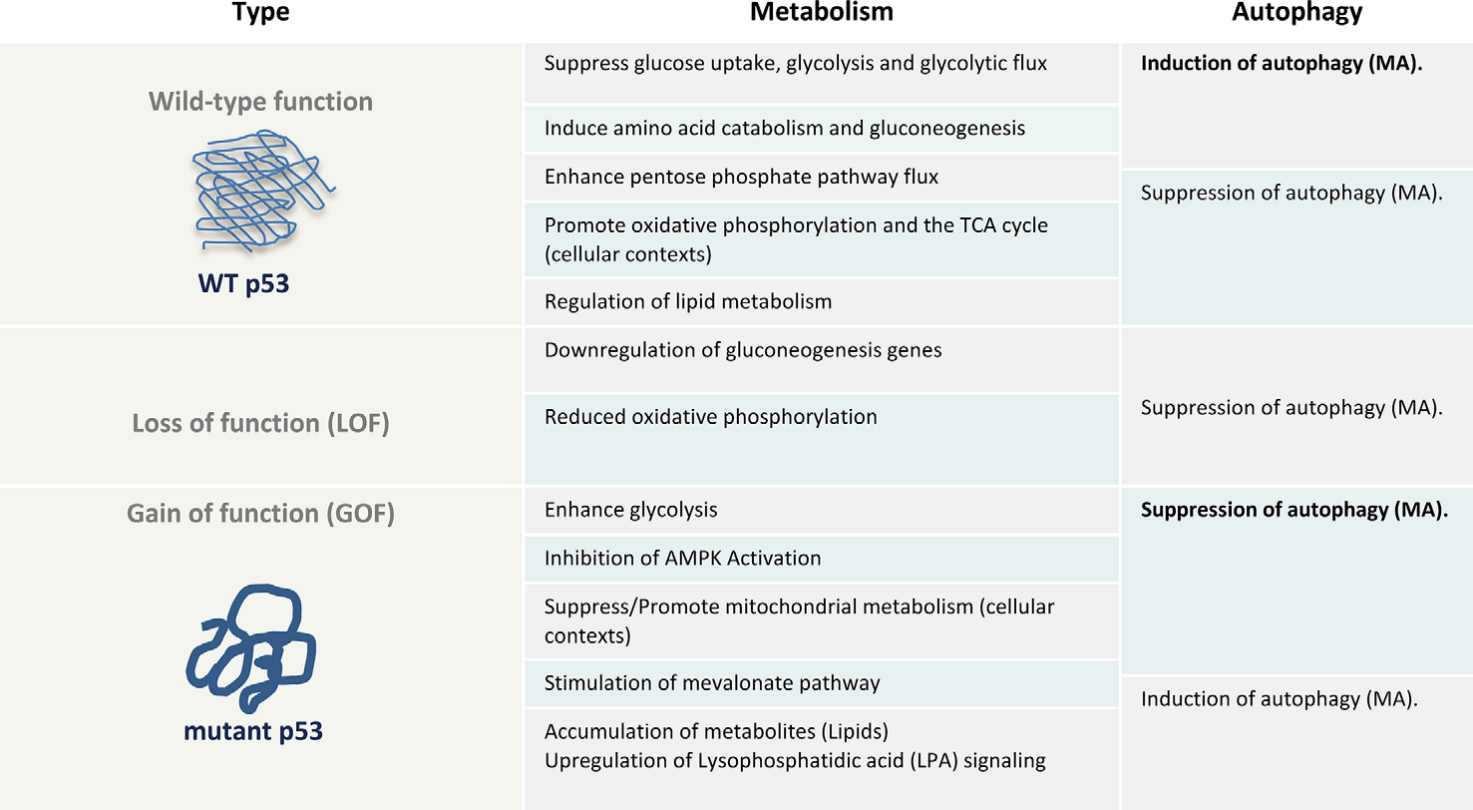
The metabolic and autophagy effects of wt p53, null (loss of function (LOF)) or gain of function (GOF) of mutant p53 as a table illustration. AMPK, AMP-activated protein kinase; MA, Macroutophagy.

While, a direct interference of p53^R175H, R273H^ mutants on MA can be denoted to LOF transcriptional repression of core autophagy genes (*BECN1*, *ATG12*) (91), most of the mutant p53-mediated autophagy inhibitory evidence stems from studies describing a gained regulatory effect of mutants on cancer metabolism (**Figure 1**). As stated above, autophagy is regulated by a number of effectors strictly interconnected with the metabolism as revealed by the fact that mTOR and AMPK are both master regulators of autophagy and major sensors of the cellular energy status (100). mTOR functions as a key homeostatic regulator of cell growth and orchestrates whether anabolic or catabolic reactions are favored. mTOR complex 1 (mTORC1) manages multiple biosynthetic pathways and promotes cell growth when nutrients are in plentiful supply. These include synthesis of amino acids, proteins and biogenesis of ribosomes (101). AMPK, on the other hand, is a highly conserved sensor of the cellular energy status that is activated upon low intracellular ATP levels. AMPK responds to energy stress by suppressing cell growth and biosynthetic processes, in part through its inhibition of the mTOR (mTORC1) pathway (102, 103). Thus, while p53 deletion and missense mutations can enhance mTOR, emphasizing the functional interplay between AMPK and wild-type p53, some mutants can display effects on the canonical AMPK-mTOR signaling beyond the transcriptional repression. An excellent example highlighting the difference between the wild-type function from null and missense GOF mutations, is the ability of p53^R175H,G245C,R282W^ mutants, displaying a negatively metabolic effects on the AMPK signaling through the direct protein-protein interaction with the AMPKα subunit under conditions of energy stress (104). Now, several p53 mutants (p53^P151S, E336X^), but not the wild-type, have been shown to interact with AMPKα through the DNA-binding domain, where mutant p53 disrupt the interaction of AMPKα-LKB1. This causes inhibited AMPKα phosphorylation and suppressed AMPK activity. In addition, a role of p53^R273H^ was demonstrated to control the mevalonate pathway (MVP) through the transcriptional modulation of SREBP1, a downstream target of AMPK (105). Furthermore, the p53^R175H, R273H^ mutants were described to promote phosphorylation on the pyruvate kinase isoform M2 (PKM2) (106), a key enzyme that catalyzes the conversion of phosphoenolpyruvate (PEP) and ADP to pyruvate and ATP in glycolysis. The phosphorylation (Tyr105) on PKM2 enhances the mTOR signaling. However, these functions seem independent of the subcellular localization of p53 mutants, as mutants with acquired capability to functionally inhibit the AMPK signaling can be found both in the cytoplasm, such as p53^P151S^, or localize exclusively in the nucleus (p53^E336X^), most likely due to the lack of a C-terminal p53 nuclear export signal, whereas the cellular localization of the mutant p53^G245C^ differs with the confluency of the cell culture. Nonetheless, based on its effect on the AMPK-mTOR axis, the cancer related expression of p53^R248W,C176S,R273H,R175H, R175H^ mutants are shown to display a gained function of affecting metabolism, thereby inhibit autophagy irrespectively of tissue of origin or prevalence to a subcellular localization (91).

Beyond the AMPK-mTOR signaling, several of the metabolic effects of mutant p53 oppose the metabolic functions commonly acquired by the wild-type protein, including glycolysis, lipid metabolism, the mevalonate pathway, *de novo* serine synthesis, urea cycle and oxidative phosphorylation (107, 108) (**Figure 1**). Thus, it is well known that mutant p53 rewires cancer metabolism (109). For example, wild-type p53 limits glycolysis and induces flux through the pentose phosphate pathway (82), whereas mutant proteins induce metabolic responses that include enhanced glycolysis to support tumor cell growth and proliferation. By promoting glucose uptake, mutant p53 can limit autophagy-dependent energy production. Therefore, any perturbation in cellular metabolism and redox control caused by p53 mutants can affect the autophagic outcome. However, this does not only apply to metabolic adaptation of cancer cells as a loss of function or in terms of enhanced glycolysis. For instance, mutant p53 has been shown to promote the MVP opposite to the wild-type p53, which is required for mutant p53 and Hsp40 interaction facilitating mutant p53 stabilization (110, 111). The MVP is an essential metabolic pathway that produces sterols and isoprenoids including cholesterol for the synthesis of membranes and lipids, as well as signal transduction allowing cancer cells to survive under conditions of matrix detachment (105). This in turn could promote detachment-induced autophagy (112). Further, beyond elevated glycolytic rate in cancer cells, several studies have clearly demonstrated that the majority of tumors similarly possess the capacity to sustain high fuel oxidation and ATP production in mitochondria (96–98, 113, 114). Especially, quiescent and slow proliferating tumor cells with activated MA, rather depend on oxidative phosphorylation for energy supply than glycolysis (98). Depending on the cellular context, mutant p53 have been indicated to both inhibit or promote oxidative phosphorylation (29, 115), and can thereby enhance or suppress autophagy. However, metabolic alterations are also observed in p53 null cells due to loss of wild type p53 function, such as a downregulation of genes that facilitate gluconeogenesis, which is observed in mice with an adipocyte-specific loss of p53 (116), and reduced oxidative phosphorylation in *p53*-null cells (117, 118). Accordingly, our understanding of the involvement of mutant p53 in direct interference of the core autophagy machinery and regulation in cancer cells as well as the detailed associated molecular mechanisms, beyond metabolic modifications, remain incomplete and need to be further assessed in human clinical specimens.

### Consideration of a Potential Stimulatory Role for Mutant p53 in Autophagy

It is equally important to note that the MA inhibitory function is not shared among all mutant p53 proteins. Mutant arising from the substitution of lysine in position 382 with arginine, fails to associate with FIP200, and loose the autophagy inhibitory function (87). Moreover, the ectopic expression of p53^P151H,R282W^ was shown not to display any efficient autophagy inhibitory behavior, apart from the fact that some mutants even show enhanced MA activities (90). Given that autophagy can sustain tumor cell metabolism, and mutant p53 can foster adaptations to nutrient deprivation, it is conceivable that certain mutant p53 proteins could therefore function in seemingly unprecedented way to respond to nutrient stress, where certain mutants may support the constitutive high levels of MA to provide selective advantage for cancer cells. Therefore, it is reasonable that some mutant p53 forms may enhance autophagy required to prevent energy crisis and maintain nucleotide pools during starvation in cancer cells caused by hypoxia and nutrition depletion in tumor microenvironment. This could be especially relevant under situations of expansion of tumor mass (**Figure 1**), in which some parts of the tumor starve due to insufficient nutrient availability or lack of vascularization, even when cancer cells promote metabolic pathways to support growth and proliferation. Under these conditions, numerous mechanisms, including autophagy activation and mutant p53 might converge to contribute to preserve cell viability as a supportive response. While this hypothesis remains speculative, a recent immunohistochemical study on 113 colorectal cancer specimens uncovered a significant association between high LC3B expression and mutant p53 protein expression pattern in ~35% of the patients (119). Although the type of p53 mutant remained undisclosed, the fact that a co-expression of LC3B and mutant p53 was tightly linked to aggressiveness is indicative of high rates of autophagy in malignant tumors. This feature was not observed in tumors with null expression. While further investigation is warranted, this finding provides the rationale that even when the wild-type ability to promote autophagy might be hampered by mutations, some mutant proteins enable autophagy activation in tumors. It is likely that certain point mutations may selectively retain some of the wild-type p53 pro-survival functions, including the pro-autophagic activity. An intriguing possibility is also that the pro-autophagic function of mutant p53 might be a transient phenotype under limited periods of nutrient starvation. A comparable example of this possibility is the activation of the cyclin-dependent kinase inhibitor p21 by p53. Although p21 expression generally contributes to the induction of an irreversible proliferative arrest, transient p53-mediated induction of p21 is reversible, allowing cells to re-enter the cell cycle once stress or damage has been resolved (120). Alternatively, the mutant p53-driven autophagy suppressive function might be overridden by additional signaling, mutations or epigenetic changes. For instance, in the context of proteasomal inhibition, cancer cells with mutant p53^R273H^ display activated MA (121). Additionally, activating mutations in *HRAS* or *KRAS* elicit excessive MA, regardless of the presence of mutant p53. In contrast to normal cells, *RAS*-driven cancer cells display remarkably high levels of basal autophagy, and it is well acknowledged that a subset of RAS-driven human cancers shows a reliance on autophagy for their survival (54). Concomitant expression of mutant p53 and oncogenic Ras, leading to cellular transformation, and a crosstalk between Ras and various mutant p53 proteins is well documented. However, in the presence of mutant p53, some *KRAS* bearing tumors are still addicted to autophagy (122), indicative of that mutant p53 may not always inhibit MA (123). Perhaps a particular pathway ultimately predominates over others. While, this remains to be investigated, it was shown that different p53 mutants cooperate with H-Ras in different ways to induce a unique expression pattern of a cancer-related gene signatures (124). For instance, the p53^R248Q, R273H^ mutants exhibited the highest level of gene expression by cooperating with NFκB, the p53^R175H^ and p53^H179R^ mutant induced the cancer-related gene signatures by elevating H-Ras activity. By contrast, the p53^G245S^ displayed no effect, further emphasizing the significantly different impact and responses different mutants can exhibit.

In addition, beyond the *in vitro* observation that even a seemingly subtle difference of one amino acid, such as p53^R248W^ versus p53^R248Q^ (125), can have a large impact on the mutant p53 function. Additionally, even the same amino acid substitutions at the same position (R175H) in the p53 protein have been shown to dramatically different phenotypic effects in terms of metabolism (29). Thus, in spite of the fact most studies describe a suppressive role of mutant p53 on autophagy, there is evidence that the contribution of individual mutant p53 on autophagy might differ in a cell or tissue type, context or cancer stage-dependent manner. Considering the tumor progression promoting function of both mutant p53 and autophagy, inhibiting autophagy seems to be counterproductive for advanced tumors. Thus, it is reasonable to think that cancer cells would rather benefit from mutant p53 with enhanced autophagy activation that can serve as a cell survival mechanism during certain conditions, similar to the dual nature of autophagy which confers suppressive role in tumor initiation while aggressive cancers acquire autophagy for growth and survival. One such condition during which mutant p53 may favor instead of counteracting autophagy is Epithelial–mesenchymal transition.

### Role of Autophagy in Mutant p53-Driven Epithelial-to-Mesenchymal Transition

One of the major transdifferentiation processes, through which cancer cells develop the ability to invade and disseminate is the Epithelial–mesenchymal transition (EMT) (126, 127). This process facilitates molecular and functional changes as such, cells undergoing EMT become invasive by acquiring characteristics required for cancer cells to adapt to phenotypic changes fostering capability to break out of the primary tumor. Beyond facilitating cancer dissemination, EMT can further contribute to stemness and resistance to therapy (128). However, EMT covers a complex and multifactorial spectrum, which gives rise to a variety of intermediate cell states (129). Consequently, EMT is now recognized as a dynamic and reversible process, rather than a binary state, that involves tumor microenvironment, cellular heterogeneities, as well as phenotypic plasticity, thus various metabolic reprograming occurs along with the EMT process.

It is well known that the EMT pathway is under the negative regulation of wild type p53, while mutant p53 proteins display oncogenic GOF activities with robust capacity to promote EMT by controlling the TGF-*β* signaling and by regulating the expression of various pro-EMT-Transcription factors (130, 131). Furthermore, considering that the metastatic potential of cancer cells increases along the EMT process, multitude of key metabolic pathways, including glycolysis, the TCA cycle, lipid and amino acid metabolism, have been attributed to contribute to EMT, tumor aggressiveness and invasiveness (132, 133). Yet, some EMT positive tumors are characterized by low proliferation rate or quiescence (134). Tumor cells with the traits of invasiveness and stemness which have undergone EMT program can manifest features of growth arrest, and cancer cell dormancy (135). The resulting stemness may drive the progression of more aggressive tumors. For instance, the TGF-*β* induced EMT process is related to a slow proliferation rate and cell cycle arrest in epithelial cells (136). This observation is rather counterintuitive as it is difficult to directly explain how a slow-proliferating population can lead to higher tumorigenicity and how these tumor cells can remain and exit dormancy.

**Figure 2:**
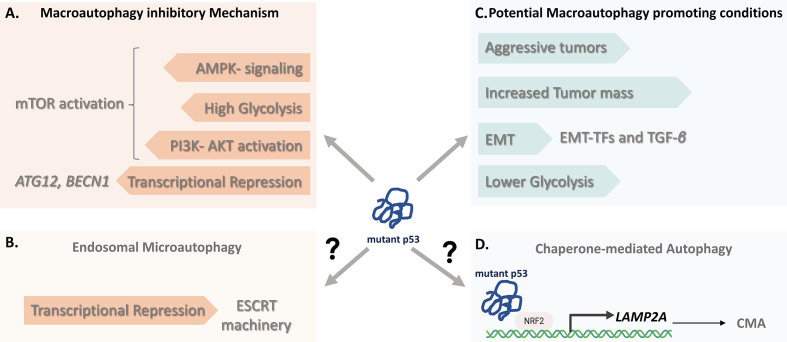
The impact of Mutant p53 on autophagy. **(A)** The macroautophagy (MA) inhibitory mechanisms of some mutant p53 proteins include the resulting transcriptional repression of core autophagy genes (*BECN1*, *ATG12*) and regulation of the mTOR activity by either constitutive blockage of AMP-activated protein kinase (AMPK) signaling or through alternative routes by affecting cancer metabolism. **(B)** Beyond MA, since wild-type p53 can transactivate genes promoting Endosomal Sorting Complex machinery, mutant p53, although yet to be determined, might negatively affect the signaling contributing to ESCRT-dependent mechanism involving endosomal microautophagy. **(C)** Cancer cells bearing some mutant p53 variants may in certain conditions favor instead of counteract autophagy. These include nutrient scarce or hypoxic conditions of aggressive tumors and with increased tumor mass, p53-mediated (EMT situations and hampering glycolysis. **(D)** LAMP-2A expression is an essential factor in CMA activation. Given that transcriptional control of LAMP-2A is shown to be under NRF2, it is likely that mutant p53 proteins might contribute to CMA activation through NRF2-mediated LAMP-2A transactivation, suggesting a molecular connection linking mutant p53 and CMA. CMA*, Chaperone-mediated Autophagy;* EMT, *Epithelial–mesenchymal transition;* ESCRT*, Endosomal Sorting Complex Required for Transport; LAMP-2A, Lysosome-associated membrane protein 2A;* NRF2, *Nuclear factor erythroid 2-related factor 2*.

One possible explanation might be that the metabolic changes elicited by mutant p53 are not mutually regulated and unidirectionally controlled in all cancer cells and may differ during the different stages of cancer progression, such as with the capacity to undergo EMT (16, 113, 137). So how could this be connected. First, several molecular mechanisms underlying the involvement of mutant p53 in malignant progression and EMT have been reported, which all converge on expansion of epithelial stem cells and induction of stem cell gene signatures, as well as mesenchymal stem cell-derived features (138–140). This suggests that mutations in *TP53* not only sustain primary tumor formation, but also that mutant p53 can promote the late stage of tumorigenesis, possibly through the acquisition of an invasive ability and stem cell characteristics. Secondly, while mutant p53 have been linked to promote glycolysis through distinct mechanisms (141), emerging data supports the notion that not all mutants display enhanced glycolysis. For example, while the p53^R175H, R273H^ mutants, are able to confer enhanced glycolysis in lung cancer cells, the stable expression of p53^R175H^ in human breast epithelial cells displayed considerably different properties, characterized by a markedly lowered glycolytic phenotype (29) (**Figure 2**). These data highlight the fact that the same amino acid substitutions, in the same position of a mutant p53 protein can have dramatically different phenotypic effects in terms of glycolysis. Moreover, breast epithelial cells expressing p53^R175H^ displayed enhanced MA, which predicts the inversely correlation between dampened glycolysis and enhanced autophagy. Thus, while mutant p53-enhanced glucose metabolism can correspondingly suppressed autophagy in proliferating cancer cells, it is reasonable that a reduced glycolysis by mutant p53 can induce autophagy in quiescent cells. Keeping in mind that a rewiring of cellular metabolism appears to precede changes in stemness, these data are supportive with metabolic changes observed in slow proliferating circulating tumor cells, which display higher mitochondrial metabolism rather than glycolysis. The observation that tumor cells with mutant p53^R72^ proteins show a greatly increased oxidative phosphorylation as well as increased metastatic ability further supports this (142).

Thirdly, we need to consider the essential role of autophagy induction in supporting cell viability during cancer progression and migration, where MA has clear positive effect on EMT (143, 144). Autophagy induction can be advantageous especially during metabolic reprogramming followed by cancer cell dormancy with a lower proliferation rate or quiescence, thus constitute an efficient adaptive strategy, which can supply of nutrients, confer stress resistance and sustain cell survival during metastatic spreading (145). Taken together, this suggest that mutant p53 may stimulate conditions of metabolic requirement for autophagy induction allowing cells to cope with a stressful or unfavorable microenvironment where cancer cells remain quiescent but may relapse (**Figure 2**). However, it is important to note that data on EMT plasticity and tumor dormancy are primarily derived from *in vitro* studies. Therefore, sophisticated animal studies are needed for tumors that have undergone mutant p53-induced EMT program to provide an *in vivo* correlate in preclinical models. Nevertheless, an important implication of these observations is that p53 mutants do not always acquire and possess the same metabolic consequences and may not display equal biological effects in all types of human cultured cells.

Therefore, when considering the generality of the effect of mutant p53 on autophagy, we might need to keep in mind the metabolic plasticity and different aspects of metabolism might be regulated in different cell or tissue types. The complex regulatory interaction between mutant p53 and autophagy might well be influenced by many factors, such as tissue and cell types, tumor stage, type of other oncogenic mutation, the sequential mutation appearance order, extent of damage or stress, and levels of intra-tumor oxygen or nutrients as well as on the proliferative capacity of the tumor cells. A switch between autophagy phenotypes, depending on fitness landscape or mutation-selection balance may as well applicable for mutant p53 and we need to consider this exceptional plasticity which might create significant challenges as we attempt to therapeutically intervene in these pathways.

### The Role of Mutant p53 in Autophagic Pathways Beyond Macroautophagy

To date, there are no direct evidence of a select mutant p53 function in microautophagy, CMA or in selective macroautophagy, including xenophagy, ribophagy. However, as wild type p53 exerts a regulatory role in mitophagy, endosome and exosome biogenesis (146, 147), it is reasonable that mutant p53 proteins might affect undiscovered functions in multiple degradative and cellular sorting systems.

The main limitations of studying non-MA pathways in regards to mutant p53 is likely the incomplete knowledge of their regulatory mechanisms. However, while the signaling mechanisms that control CMA are currently not fully understood, a key step in the CMA process is the expression of LAMP-2A receptor at the lysosomal membrane. High lysosomal LAMP-2A levels are reported to correlate with a predisposition of CMA, whereas silencing of LAMP-2A results in inability to degrade proteins *via* the CMA pathway, thus increase LAMP-2A expression is an essential factor in CMA activation (148). Accordingly, a transcriptional control of LAMP-2A expression is shown to be under the control of the NFE2L2/NRF2 (Nuclear factor erythroid 2-related factor 2 (NRF2) (149), also known as nuclear factor erythroid-derived 2-like 2 (NFE2L2), which generally participates in the control of metabolic redox processes including degradation of oxidized proteins. In 2018, the missense mutant variant p53^R280K^ was demonstrated to interact with NRF2 and to contribute to selective activation of its downstream transcriptional program (150). Thus, given that NRF2 promotes a pro-survival oxidative stress response that allow cells to cope with oxidative stress, along with the fact that CMA is induced by oxidative stress, it is likely that mutant p53 proteins might contribute to CMA activation through NRF2-mediated LAMP-2A transactivation, indicative of a molecular pathway that connects mutant p53 with CMA (**Figure 2**). Consistent with this indication, analysis of various human cancer cells with different mutational p53 status that either expressed wild-type, mutant p53 or null in p53 expression, revealed that Spautin-1 induced CMA in confluent growth conditions selectively induced cell death of mutant p53-expressing cancer cells. No or little effect was detected in wild-type p53 or p53-null cancer cells, suggesting that cancer cells with mutant p53 might be more susceptible to activate or undergo CMA (65).

Moreover, considering that wild-type p53 can transcribe several critical genes encoding endosomal compartment, including *TSAP6*, *CHMP4C* and *CAV1* (147), provides a rationale that p53 signaling may contribute to Endosomal Sorting Complex Required for Transport (ESCRT) machinery dependent mechanism, involving endosomal microautophagy (eMI). However, it is yet to be determined whether there is an involvement of mutant p53 proteins in micro- or endosomal microautophagy (**Figure 2**).

Furthermore, beyond autophagic pathways, mutant p53^R273H^ has been shown to drive alterations in endocytic membrane trafficking during which DNM1 and Myosin VI (Myo6) were upregulated in cancer cells. Apart from stimulating the expression of endosomal proteins, both the wild-type and p53^R273H^ mutants are indicated to effect proteins involved in the secretory pathway, protein secretion *via* extracellular vesicles (EV) and exosomes of endosomal origin (151). Thus, mutant p53 might regulate the expression of components of the endocytic machinery and modify secretion of extracellular vesicles in multiple ways.

## Mutant p53 as Target of Autophagy

### Targeting Mutant p53 Proteins

Based on the high frequency of *TP53* mutations in human tumors, the oncogenic effects of many missense variants with the fact that cancer-specific pathogenic stabilization of mutant proteins effectively sustains tumor progression and dissemination, mutant p53 proteins represent indisputable promising targets in cancer therapy (17, 152). Accordingly, different approaches have been explored in which targeting of mutant p53 has primarily focused on the development of therapies designed to inhibit the mutants and restore their wild-type p53 function by small molecules, targeting the gain-of-function phenotype of mutants and stimulating immunological activity directed against a mutant p53 protein (153, 154). In animal models, targeting mutant p53 functions have been shown with highly promising results that selectively kill cancer cells, with low toxicity in healthy tissues, indicating tumor-specific vulnerabilities (105, 155, 156). But in the clinics, the specific targeting of mutant p53 proteins has proven challenging, especially considering that mutations are diverse in their type, sequence context, position, and structural impact, making it difficult to identify a well-defined structure (2, 7). In fact, most desirable oncoprotein targets in cancer therapy, including mutant p53, belong to the intrinsically disordered proteins, which lack a well-defined protein structure making them challenging to pharmacologically target (157). An important factor in anticancer therapeutic failure is also associated with pharmacologic drugs that may lack response to all mutant variant or with substantial toxicity due to loss of wild-type function, or activating wildtype in normal tissue. Thus, while targeting a loss of function is difficult, growing evidence indicate that no single drug may display equal impact on all mutant proteins. Development of different drugs to target distinct mutant p53 or their activities is therefore time consuming and not cost-efficient, although such drugs could make a huge impact. Hence, exploring of alternative approaches to target mutant p53 proteins is therefore of high importance.

During recent years strategies of stimulating the cell’s own quality control mechanisms to prevent the aberrant accumulation and induce degradation of oncogenic proteins, including mutant p53, are being explored as a new therapeutic approach. Central to this idea is that oncogenic mutant p53 functions and the mutant p53 addiction of cancer cells is reliant on its sustained high levels, thus this addiction can be therapeutically exploited by targeted mutant p53 degradation strategies. Beyond pharmacological blockade of mutant p53 stabilizing mechanism to promote proteasome-dependent proteolysis, the considerable role for targeted degradation into lysosomes is suggested as a new advance to have a potentially major impact on mutant p53. The targeting of mutant p53 proteins by autophagy activation could offer promising future therapeutic option and is therefore currently investigated intensively. Below, we describe recent advances strategies that and might be potential therapeutic methods.

### Targeting Mutant p53 by Macroautophagy

**Figure 3:**
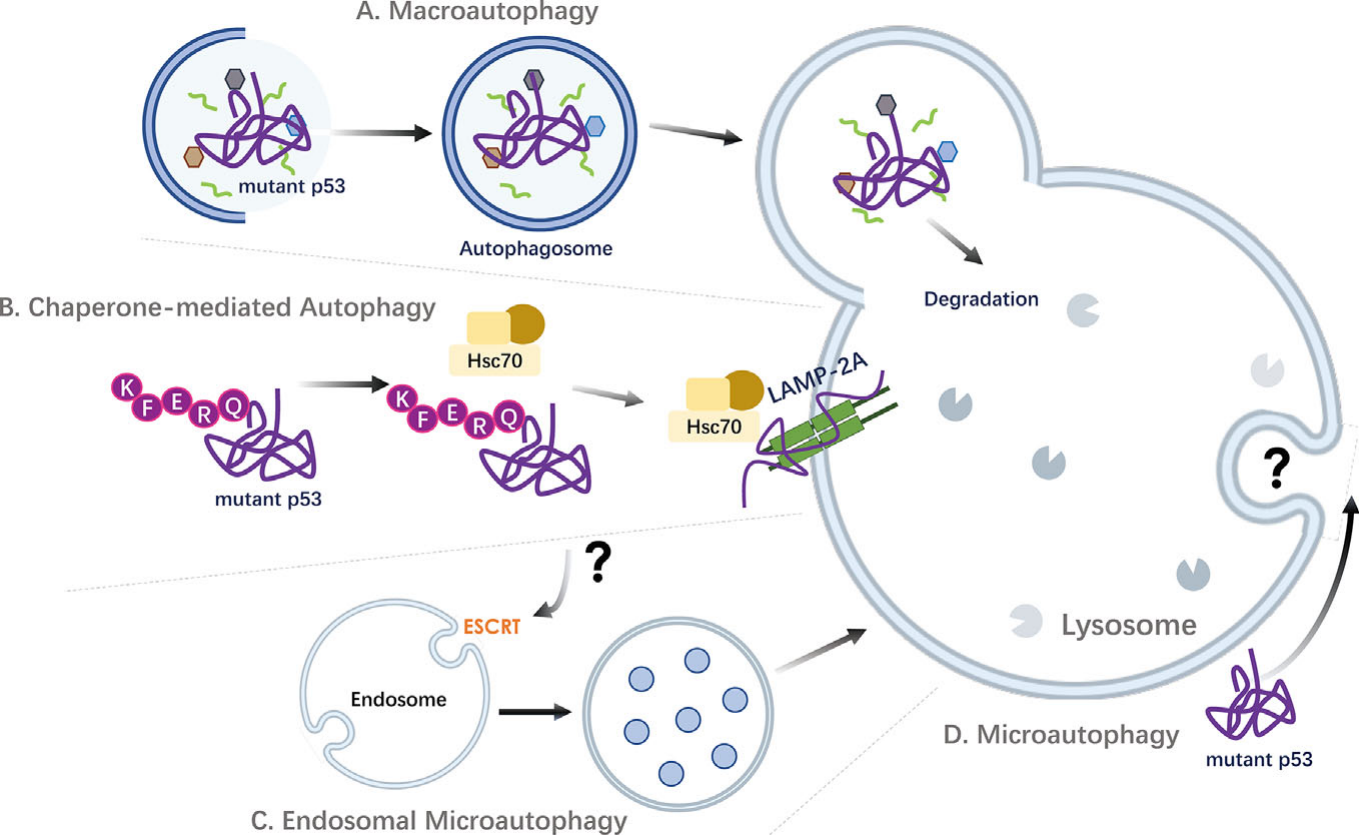
Targeting mutant p53 by autophagic pathways. Strategies of autophagic degradation of accumulated oncogenic mutant p53 proteins in cancer cells. **(A)** Mutant p53 can be engulfed and degraded *via* macroautophagy. P53 containing aggregates have also been implicated to undergo degradation by aggrephagy, a selective sequestration of protein aggregates by macroautophagy. **(B)** As p53 contains KFERQ-like motifs, mutant p53 proteins can be targeted and degraded through the stimulation of Chaperone-mediated Autophagy (CMA). **(C)** *Via* the recognition of proteins harboring KFERQ-like motifs, the molecular chaperone HSC70 and co-chaperone complex can also promote the localization of cargo proteins into endosomal compartments in an ESCRT machinery dependent mechanism, through a process called endosomal microautophagy (eMI). Thus, beyond CMA, other autophagic pathways, including endosomal eMI may mediate the degradation of mutant p53. **(D)** The direct uptake of mutant p53 proteins by lysosomes through microautophagy is not known. ESCRT, *Endosomal Sorting Complex Required for Transport;* Hsc70*, Heat shock cognate 71 kDa protein (also known as HSPA8);* LAMP-2A*, Lysosome-associated membrane protein 2A*.

Although mutant p53 proteins were known to accumulate at abnormally high levels in cancer cells, the observation that lysosomal inhibitors could further stabilize mutant protein abundances strongly implied that they might be continuously degraded through the lysosomal pathway. In line with this, glucose restriction in multiple cancer types bearing the p53^R175H, R280K^ mutants was shown to induce p53 mutant deacetylation, routing it for degradation *via* MA (158) (**Figure 3**). Accordingly, several studies now demonstrate that lysosomes indeed represent a degradation route for certain mutant p53 proteins (159–161). MA inhibition by either chemical inhibitors or downregulation of key autophagic related genes (*ULK1*, *BCN1* or *ATG5*) induce stabilization of mutant p53, while, the overexpression of Ulk1 or Beclin-1 results in mutant p53 degradation (162). With MA as an emerging important pathway involved in the stability of mutant p53, several classes of small molecules enabling efficient mutant p53 degradation through the induction of autophagy has been described. These include, a) the curcumin-based zinc compound (Zn(II)-curcumin and capsaicin (8-methyl-N-vanillyl-6-noneamide)-induced macroautophagy which have been shown to deplete the expression of p53^RH175^ and p53^R273H^ mutants (159, 163), b) Gambogic acid, a pro-apoptotic molecule that promotes the p53^R280K^ and p53^S241F^ mutant degradation by inducing autophagy (160), c) inhibition of MKK3, a dual protein MAP kinase, which reduces p53^R273H^ mutant protein levels through ER stress-induced autophagy, d) the cruciferous-vegetable-derived phenethyl isothiocyanate (PEITC), which render the p53^R175H,R273H, R248Q^ mutants by degradation following reactivation of the mutants, e) heat shock protein 90 (HSP90) inhibitors such as 17-allylamino-17-demethoxygeldanamycin (17-AAG) or ganetespib (155), and f) histone deacetylases inhibitors (HDACi), which have been studied as anticancer compounds based on their potential to stimulate autophagy and to degrade p53^R172H,R248Q, R280K^ mutants (155, 164–167). Although apoptosis seems as the main route, inhibiting the HDACs, for example by the suberoylanilide hydroxamic acid (SAHA), a pan HDAC inhibitor, is shown to induce the destabilization of the HDAC6–HSP90–mutp53 complex (165), that results in mutant p53 degradation in cancer cells with pronounced autophagy induction, such as in MDA-MB-231 bearing the mutant p53^R280K^ (164). However, while the MA stimulatory effect of SAHA on cancer cells carrying mutant p53 has been suggested, compared to null or wild-type p53 expressing cells, DLD1 cells carrying the p53^S241F^ allele was not affected by this action. The observed degradation of p53^S241F^ proteins upon SAHA exposure was suggested to relate on alternative degradation pathways rather than MA. Yet, ES2 cell lines bearing the same mutant (p53^S241F^) show difference in SAHA sensitivity. This strongly suggests that, cell type and -contexts need to be considered for SAHA-mediated cytotoxicity in cancer cells. Further, the Zn(II)-curcumin and capsaicin by acting on protein folding is able to reactivate wild-type p53 that induces its target gene DRAM to promote autophagy, while gambogic acid is shown to induce mutant p53 protein degradation through proteasome ubiquitination by carboxy terminus of Hsc70 interacting protein (CHIP). CHIP, on the other hand, known to display chaperone and E3 ligase activity, is involved in stabilizing and degrading both wild‐type and mutant p53 proteins, where the degradation of mutant p53 by CHIP was shown to be *via* autophagy through K63‐linked polyubiquitination. Under both normal and hypoxic conditions CHIP was shown to selectively degrade aggregation‐prone mutants p53^R110L^, p53^R110P^ and p53^R175H^, without significant effects on the level of nonaggregating mutant p53^R248W^ and p53^R273H^ (168). Moreover, the p53^R175^ degradation by PEITC was reported to be mediated by both the proteasome and autophagy in a concentration-dependent manner, underlying the importance and need for further investigations for the selective degradation mechanism of mutant p53 in order to develop selective autophagy targeting therapeutic strategies. In addition, it is important to note that while the wild type p53 proteins are directed for proteasome-dependent degradation, autophagy-lysosome degradation is also attributed to control cellular p53 stability (169, 170). For example, Sunitinib, a small molecule multi kinase inhibitor, approved for the treatment of metastatic renal cell carcinoma, induced autophagic degradation of wild type p53 proteins in multiple cancer cell lines (169). However, the molecular mechanisms and cellular players involved in autophagic degradation of wild type p53 are still not fully known.

### Mutant p53 Proteins as Targets for Chaperone-Mediated Autophagy

Beyond contributing in lysosomal degradation of a select subset of cellular proteins, the discovery of mutant p53 proteins as CMA targets established a regulatory role for CMA in oncoprotein degradation and its potential tumor suppressive role (37, 65, 67, 171). Thus, a new degradative detour for mutant p53 *via* CMA was uncovered (172) (**Figure 3**).

As previously mentioned, CMA is a unique type of mammalian autophagy that only applies to select proteins without targeting cellular organelles (42). Its specificity relies on the recognition of a pentapeptide CMA motif (KFERQ-like) that is a prerequisite in target proteins. The cytosolic heat-shock cognate protein of 70 kDa (Hsc70/HSPA8) plays an essential role in CMA by recognizing the KFERQ-like sequence motifs in substrate proteins. Indeed, p53 harbors two pentapeptide sequences (_200_NLRVE_204_ and _341_FRELN_345_) that are consistent with an Hsc70 recognition motif (65). The FRELN motif is on the linker region, while the _200_NLRVE_204_ motif is exposed on the surface of the p53 protein, making it accessible for recognition.

Once activated, CMA was shown to be very effective in degrading different mutant p53 proteins, regardless of their mutational status (65), including p53^P98S,P151H, A161T,R175C,R175D,R175H,L194F,S227K,S227R,G245C,R248L,R248W,E258K,R273H,R273L,R280K, R282W^. This was initially illustrated by the assessment of CMA activation on the ectopically overexpressed above mentioned p53 mutants in a p53 null colon cancer cells. Subsequently, the CMA-mediated degradation of cancer associated endogenous mutant p53 proteins was shown on p53^R175H,R248Q,S241F,R158InF,R280L,G266Q^ variants (65). This suggest that CMA-mediated mutant p53 degradation may be more efficacious than treatment with targeted mutant p53 specific reactivating small molecules and that CMA-based strategy could overcome resistance from acquired mutations. Importantly, the activation of CMA was not or less effective on wild type or p53 null expressing cancer cells. However, contrary to cancer cells, hepatitis C virus infection induced ER-stress response, which leads to CMA stimulation in untransformed primary human hepatocytes results in degradation of wild type p53 (173). The increased expression of chaperones due to unfolded protein response and ER stress associated with the CMA response, where the genetic silencing of *LAMP-2A* restored the observed p53 degradation. In fact, the silencing of *LAMP-2A* under irradiation conditions was also shown to result in increased p53 protein level (174), in some cancer cells, such as HepG2, which expresses wild type p53 (173). While these studies suggest an interplay between the CMA pathway and wild type p53, it should be kept in mind that p53 interacts with a wide range of different proteins, thus the accumulation of p53 upon LAMP-2A knockdown, may therefore depend on recognition of its molecular partners by the CMA pathway, such as HMGB1 degradation with further impact the wild type p53 protein expression (174). Nonetheless, the discovery that mutant p53 proteins are CMA substrates provided experimental evidence that CMA could be exploited as a novel approach to eliminate mutant p53 in cancer cells. Accumulating evidence now support that CMA activation plays a role in mutant p53 targeting (161). In fact, beyond mutant p53, CMA has been shown to promote the degradation of other oncoproteins, as HK2 and c-Myc (66, 67). Further, a decrease in CMA with age has been associated with higher risk of malignant transformation, and mice with hepatic blockage of CMA has been shown to develop spontaneous tumors (68). While, these findings suggest clinical implications of CMA activation, to date the role of CMA in tumorigenic conditions is not well-defined and there are no direct pharmacological CMA activators for cancer cells. Characterization of such activators would also require that it does not affect other degradation pathways. Accordingly, in order to explore the clinical implementation of CMA, development of applicable methods to measure CMA in live cells, and *in vivo* studies in CMA activation is needed. Thus, to date, there are no clinical studies launched to demonstrate the efficacy of CMA activation in patients. However, the knowledge of the its oncogenic targets, such as mutant p53, and understanding its selective degradation mechanism is an excellent starting point for future development of targeted therapeutic strategies involving CMA.

### Mutant p53 as Possible Target for Microautophagy and CASA

Beyond CMA, recognition of proteins harboring a KFERQ-like motif by the molecular chaperone HSC70 can also lead to the endosomal localization in an ESCRT machinery dependent mechanism, through a process called endosomal microautophagy (eMI) (40, 47). Thus, selective degradation of single proteins has been described in a HSC70-driven endosomal eMI pathways (40, 42, 175). In addition, chaperone-assisted lysosomal degradation pathway CASA (chaperone-assisted selective autophagy), has been reported to require the involvement of HSC70. Keeping in mind that the amino acid sequence of p53 contains KFERQ-like motifs that is recognizable by HSC70, although yet to be proven, it is plausible that mutant p53 protein might be targeted by eMI or CASA (**Figure 3**). However, it is currently not known whether wild type and/or mutant p53 proteins are targeted and degraded by these pathways.

### Mutant p53 Aggregates as Target for Aggreaphagy

While accumulation of protein aggregates is commonly known for their involvement in the onset of many neurodegenerative diseases, the conformation of mutant p53 with missense mutations is now known to share similarity with that of pathological mutant proteins involved in a wide range of neurodegeneration, including Alzheimer disease, Parkinson disease, and amyotrophic lateral sclerosis, the so-called protein conformation diseases that involve protein misfolding in their etiology (176). Accordingly, mutant p53 proteins display hyperstability due to acquired misfolding and partially denatured conformation with high tendency to form amyloid like micro- and macro-aggregates both *in vitro* and *in vivo* (177). The aggregation of mutant p53 (amyloid oligomers and fibrils) confers a prion-like activity on the native protein, converting it into an inactive form, thus contribute to its oncogenic function (178). The formation of aggregates largely depends on cellular chaperones and chaperone-assisted proteins. Accordingly, mutant p53 stabilization is achieved by the interaction with chaperone heat shock proteins (HSP), including HSP90, HSP40 and HSP70, that cooperate in stabilizing mutant p53.

Aggregated proteins can be degraded by the proteasome or CMA, however, only after the dissolution into soluble single peptide species, unless targeted by a process called aggrephagy, a selective sequestration of protein aggregates by macroautophagy (179). While the molecular mechanism of cargo selection during aggrephagy needs to be further elucidated, p53^R175^ containing aggregates have been implicated to undergo degradation by this pathway (167). This is in fact in line with the observations that CHIP, beyond targeting wild‐type p53 by K48 polyubiquitinition, preferentially degrades aggregation-prone mutant p53 proteins through K63 polyubiquitinition chains (168). Thus, although accumulation of mutant p53 occurs only in cancer cells, in which most missense mutants are shown to be more stable than wild-type p53, the aggregation of different mutants seems to correlate with individual structural characteristics, which may affect their differential recognition and degradation route.

### Degradation of Mutant p53 Proteins by Multiple Autophagic Pathways

Autophagy pathways are mechanistically and functionally linked such that blockage to either one can lead to upregulation of the other in a way. The degradation of distinct mutant variant can therefore vary between the different types of autophagy, when one pathway is blocked or inhibited, or in response to different stresses. However, it is important to note that although MA and CMA are both operational under normal nutritional conditions, their basal activities are not sufficient for efficient removal of mutant p53. Rather, as described above, mutant proteins can undergo degradation through MA induced by glucose restriction or by proteasomal inhibition, however when MA is inhibited, which significantly accelerates the activation of CMA that in turn promotes the degradation of mutant p53. This differential degradation route was demonstrated for the p53^R248Q^ mutant in a context dependent manner. In tumors growing in normoxia, with no stress, the treatment with Hsp90 inhibitor (17-AAG) was able to induce the degradation of p53^R248Q^ through MA (161). However, during metabolic stress caused by the pyruvate dehydrogenase kinase-1 (PDK1) inhibitor dichloroacetate (DCA), p53^R248Q^ proteins were stabilized by increased interaction with the Hsp90 chaperone machinery. Thus, in this condition, the co-treatment of 17-AAG instead promotes the association of p53^R248Q^ with Hsc70 and CMA activation, resulting in p53^R248Q^ degradation *via* the CMA pathway (161). Thus, different metabolic contexts and stressors induce diverse autophagy mechanisms that can degrade mutant proteins. In fact, beyond enabling efficient p53^R248Q^ degradation by either MA or CMA, the HSP90 inhibitor, geldanamycin, has been suggested with an unspecific ability to activate CMA.

Furthermore, it is unclear whether different autophagic pathways may display any preference to degrade certain mutants. Since mutant p53 proteins encoded by different mutant alleles exhibit a distinctive tendency to misfold and aggregate, it may affect their susceptibility for recognition and targetability, thus it is reasonable that the mutational status may play a determinant role in its ability to be degraded through the distinct autophagic system. For instance, this may be due to the diverse ability of certain mutant to aggregates into prion-like amyloid oligomers, including p53^R175H, R249S^, which can form larger multimeric assemblies, while p53^R248Q^ mutant displays significantly increased amyloidogenic potential, whereas p53^M237I^ mutant is shown to co-localize with amyloid oligomers (180). Thus, beyond defects in degradation and recognition mechanisms, the accumulation of mutant p53 proteins to different levels in cancer cells may depend on their targetability by multiple vs certain degradation pathways.

## Concluding Remarks

To conclude, the role and impact of mutant p53 in autophagy regulation is complex, context-dependent and far from fully elucidated. Growing evidence along with rapidly developing genome editing and omics techniques are likely to revolutionize new roles and autophagic activities of different mutant p53 proteins that may vary according to changes within tumors or in the tumor microenvironment. These new technologies may shed new insights for a knowledge-based discovery to identify knowledge gaps and analyze scenarios that require a reconsideration for the function of mutant p53 on autophagy.

Further, since it is clearly demonstrated that mutant p53 stabilization is a tumor-specific vulnerability, strategies to promote the degradation of mutant p53 by autophagy represents an attractive anti-cancer approach. Yet the effective therapeutic use of autophagy induction requires detailed knowledge of how the autophagy-lysosome pathway might be affected in cancer diseases. This is especially important given that disease-related genetic defects may affect autophagic pathway e.g., when lysosomal fusion or degradation is impaired. Thus, the stimulation of autophagy may rather worsen the disease progression. While autophagy modulation is an exciting area of clinical development, the effects of autophagy upregulation may vary substantially depending on the precise nature of the tumor state. Further comprehensive understanding of the roles of autophagic pathways throughout different stages of carcinogenesis has potential to guide development of novel therapeutic strategies to eradicate cancer cells with mutant p53. Furthermore, most if not all autophagy modulating drugs in clinical trials are inhibitors of the process, with the effectiveness of inhibiting autophagy to enhance chemotherapy cytotoxicity. Accordingly, pharmacological methods are not currently available to selectively and solely activate and target oncoproteins, including mutant p53, by autophagic pathways. While CMA can be directed to target oncogenic proteins, such as mutant p53, molecular mechanisms of its selective cargo recognition remain largely uncharacterized.

## Author Contributions

All authors listed have made a substantial, direct and intellectual contribution to the review and writing, and approved it for publication.

## Funding

This work was supported by grants from Karolinska Institutet, the Swedish Research Council (VR), the Ragnar Söderberg Foundation and the Swedish Cancer Society (Cancerfonden). Special thanks to Dr. Zhang for the graphic design of figures.

## Conflict of Interest

The authors declare that the research was conducted in the absence of any commercial or financial relationships that could be construed as a potential conflict of interest.

## Abbreviations

AMPK, AMP-activated protein kinase; CDKN1A, Cyclin Dependent Kinase Inhibitor 1A; Chmp4C, Charged multivesicular body protein 4c; CMA, chaperon-mediated autophagy; DAPK1, Death-Associated Protein Kinase 1; DNM1, Dynamin 1; DRAM1, damage-regulated autophagy modulator 1; EGFR, epidermal growth factor receptor; EMT, Epithelial–mesenchymal transition; ESCRT, endosomal sorting complexes required for transport; GOF, gain of function; HIF-1, hypoxia inducible factor 1; IGF-BP3, Insulin-like growth factor-binding protein 3; IGFR, Insulin-like growth factor receptor; LAMP-2A, lysosome-associated membrane protein 2A; LKB1, liver kinase B1; MDM2, murine double minute 2; MKK3, Mitogen-activated protein kinase kinase 3; mTOR, mechanistic target of rapamycin; PI3K, phosphatidylinositol 3-kinase; PKM2, pyruvate kinase isoform M2; RB1CC1, Retinoblastoma coiled coil protein 1; REDD1, protein regulated in development and DNA damage responses 1; ROS, reactive oxygen species; SREBP1, sterol regulatory element-binding protein 1; TGFBR, transforming growth factor-beta receptor; TIGAR, TP53-induced glycolysis and apoptosis regulator; TSAP6, tumor suppressor-activated pathway 6; TSC, Tuberous Sclerosis Complex.
